# Mechanical Performance of Cellulose Nanocrystal and Bioceramic-Based Composites for Surgical Training

**DOI:** 10.3390/polym16192849

**Published:** 2024-10-09

**Authors:** Hee-Chang Jeon, Young-Seong Kim

**Affiliations:** 1 Quantum Functional Semiconductor Research Center, Dongguk University, Jung-gu, Seoul 04620, Republic of Korea; hcjeon@dongguk.edu; 2 Department of Mechanical, Robotics and Energy Engineering, Dongguk University, Jung-gu, Seoul 04620, Republic of Korea

**Keywords:** orthopedic surgery, finite element method, fracture toughness, bioceramics, cellulose nanocrystal (CNC)

## Abstract

This study evaluated the mechanical performance of a cellulose nanocrystal (CNC)-based composite, consisting of hydroxyapatite and natural fibers, mimicking the mechanical properties of real bone. The effect of natural nanofibers on the cutting force of the composite was evaluated for suitability in surgical training. Although hydroxyapatite has been extensively studied in bone-related applications, the exploration of epoxy-based composites incorporating both hydroxyapatite and CNC represents a novel approach. The evaluation involved a load cell with an oscillating saw. The uniform distribution of CNCs within the composite was assessed using 3D X-ray imaging. The cutting force was found to be 4.005 ± 0.5469 N at a feed rate of 0.5 mm/s, comparable to that required when cutting real bone with the osteon at 90°. The 90-degree orientation of the osteon aligns with the cutting direction of the oscillating saw when performing knee replacements on the tibia and femur bones. The addition of CNCs resulted in changes in fracture toughness, leading to increased material fragmentation and surface irregularities. Furthermore, the change in the cutting force with depth was similar to that of real bone. The developed composite material enables bone-cutting surgeries using bioceramics and natural fibers without the risks associated with cadavers or synthetic fibers. Mold-based computed tomography data allows for the creation of various bone forms, enhancing skill development for surgeons.

## 1. Introduction

Surgeries involving bone cutting or drilling can exhibit significant individual variability. Studies have shown that practice can improve the surgical skills required for these procedures [1]. Surgeons face difficulties in precise control owing to the cutting force and blade bending, which subsequently affect the feed rate [2]. Moreover, the postoperative condition of the implant surface is often suboptimal [3].

Research on bone-cutting force began in 1974, with a focus on the factors influencing the cutting force through orthogonal cutting [4]. Studies have examined the tool rake angle and depth of the cut, exploring chip and crack formation in bones, which has led to the development of new tools to reduce the cutting force; however, most of these previous studies utilized rotational cutting tools [5]. An oscillating saw has been developed to reduce the cutting force [6], and the saw blades currently used in orthopedic surgery have been redesigned with teeth. The tooth shape of the blade is crucial during the oscillation process, prompting various research directions to minimize the cutting force, leading to the study of cutting force models based on oscillating saws [7].

Surgical oscillating saws are widely used in orthopedic surgery to cut various hard tissues, particularly in total knee arthroplasty [8]. These hard tissues, including the cortical bone, cancellous bone, dentin, and enamel, have complex anisotropic structures, resembling composites, and exhibit significant individual variability [9]. Understanding the cutting mechanism of hard tissue is essential for surgeons to improve the machining process [10,11]; enhance various surgical cutting, milling, and drilling procedures; and use appropriate tools [12,13]. Cadaver bones are typically used for surgical education and training; however, they pose challenges such as cost, limitations in practice, and preservation issues. Furthermore, the aerosols generated during the machining of cadaver bones can act as pathogen carriers, posing health risks when inhaled [14].

Regarding research materials for bone-cutting training, epoxy-based composites have been explored as alternative materials, offering mechanical properties similar to human bone. Research efforts are underway to develop composites with bone-like mechanical properties for medical applications. However, despite their excellent biocompatibility and bone repair properties, they have significantly lower mechanical strength compared to epoxy-based composites and are challenging to fabricate into femur shapes [15,16]. Poly lactic acid (PLA)/hydroxyapatite composites have been studied for bone modeling, with the material fabricated into filaments and used to create bone shapes via 3D printing. Additionally, PLA-based alginate–hydroxyapatite composites have been investigated for application to femur bones. However, these materials face limitations in producing full-scale femur models, and their compressive strength and elastic modulus are significantly lower than natural bone [17,18]. Epoxy resin can replicate various cortical bone shapes using molds and maintains its post-curing form, making it suitable for producing identical bone models. Therefore, there is growing interest in creating safe, cost-effective, and mechanically and geometrically similar bone models for traditional surgical education [19]. However, there are some limitations to these bone models; the synthetic fibers (such as carbon and glass) used for cutting can generate dust that may cause dermatitis in practitioners, and prolonged exposure to synthetic fiber dust has been shown to negatively affect respiratory health [20,21,22,23]. In contrast, nanocellulose is a safe material used in the field of biomedicine and possesses biocompatibility characteristics [24]. Additionally, nanocellulose is widely used in polymer-based composites, similar to hydroxyapatite (HAP) [25].

This study is the first to analyze the oscillating force on composite materials and investigate the cutting-force characteristics of a novel nanocellulose-based epoxy composite with fracture toughness. Previous studies have shown that increased stiffness due to nanocellulose results in higher cutting-force load. Furthermore, for the first time, the distribution of nanocellulose within the composite was analyzed using three-dimensional (3D) X-ray imaging.

## 2. Materials and Methods

The epoxy and hardener used were 105 epoxy resins and 207 hardeners (West System Co., Bay City, MI, USA). HAP was obtained from Sigma–Aldrich (St. Louis, MO, USA). The nanocellulose used in this study consisted of cellulose nanocrystals (CNCs) provided by the Process Development Center at the University of Maine. Silicone molds were created using Mold Max 30 (Smooth-On, Inc., Macungie, PA, USA) for specimen preparation. The oscillating saw used was a DPS model from Sungwon Medical (Chungbuk, Republic of Korea) with saw blade dimensions of 90 × 25.4 × 1.27 mm^3^. The angle of the tooth was measured to be 51.8 ± 1.2°.

### 2.1. Preparation of Nanocellulose

A CNC slurry with a concentration of 10.4 wt% was freeze-dried at −86 °C using a freeze dryer (ilShinBioBase Co., Ltd., Dongducheon-si, Republic of Korea, FD8508) for 3 days to obtain a powder.

### 2.2. Preparation of Composite Materials

First, the HAP was vacuum dried at 100 °C for 30 min. The HAP was measured according to its weight percentages and dried accordingly. After drying, the hardener was added, and the mixture was ultrasonicated for 30 min to ensure thorough mixing of the hardener and HAP powder. CNCs were added to the hardener and HAP mixture and subjected to ultrasonic treatment together. Following ultrasonication, the resin was poured into the mixture and stirred with a wooden stick. To remove bubbles, the mixture was subjected to vacuum treatment at room temperature. The prepared mixture was then poured into silicone molds of the desired specimen shape and cured. The curing process was completed by heating at 80 °C for 3 h, followed by physical property testing. The hardener and resin were mixed at a weight ratio of 1:3. This method and the mixing ratio follow the same manufacturing process as described in the previous study [26] (Table 1).

Single-edge-notch bending (SENB) method specimens were fabricated according to the ASTM D5045 standard [27], with dimensions of B (Thickness) = 5 mm, W (Width) = 10 mm, L (Length) = 44 mm, and a (Length of the notch) = 0.45 mm.

### 2.3. Material Characterization

A Universal Testing Machine (UTM; Oriental TM Co., Ltd., Siheung-si, Republic of Korea, OTU-2) was used for mechanical testing. A load cell and a vice were installed on the upper part of the machine. The cuboidal composite material blocks were fixed in the vice to keep them steady. A custom-made oscillating saw was installed and secured to the lower part of the UTM. The cutting force was measured by adjusting the feed rate of the UTM at a temperature of 24 °C. All tests were conducted with three measurements of the cutting force.

Fracture toughness was measured using the SENB method following the ASTM D5045 standard. The experiment was conducted with a feed rate of 0.05 mm/min.

For the internal structural analysis of the nanocellulose-reinforced composite, X-ray microscopy (XRM) was performed using the Xradia 620 Versa (ZEISS, Baden-Württemberg, Germany) installed at the National Center for Inter-university Research Facilities (NCIRF) at Seoul National University. Dragonfly software (version 2024.1 for Windows, Comet Technologies Canada Inc., Montreal, QC, Canada) was used to analyze the nanocellulose volume fraction and particle distribution within the composite material as well as the XRM data.

### 2.4. Finite Element Method

Crack growth was simulated using the eXtended finite element method (XFEM) based on the 3D model of SENB specimens, with the experimental material properties of each specimen implemented. The simulation was performed using the Abaqus/CAE 2020 software.

### 2.5. Bone Model Fabrication

To create the bone model, a 3D printer (DP201, Sindoh, Seoul, Republic of Korea) was used to print the shape of a femur bone using polylactic acid material. The printing speed was set at 40 mm/s, with a nozzle temperature of 200 °C and a bed temperature of 25 °C (room temperature). A 15 mm raft was used to secure the print to the bed. The printed 3D model was coated with XTC-3D epoxy resin (Smooth-On, Inc.). The acrylic and bone model was then set up, and Mold Max 30 silicone (Smooth-On, Inc.) was poured to create the mold. Using a process identical to composite material manufacturing, the material was poured into the mold and cured to produce the composite bone model.

## 3. Results and Discussion

**Figure 1:**
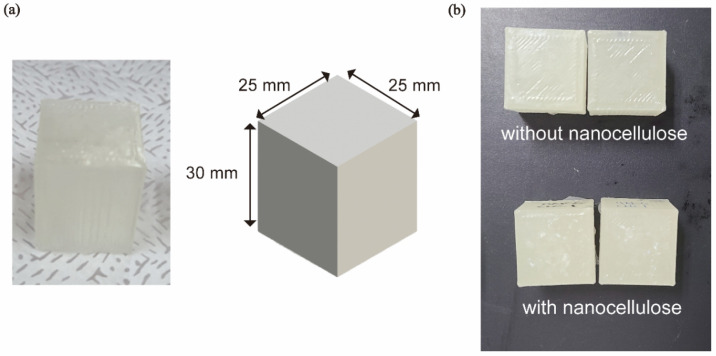
Preparation of specimens for measuring the cutting force. (**a**) The shape of the specimen fabricated in a cuboid form, with dimensions 25 × 25 × 30 mm. (**b**) Cuboid specimens made using hydroxyapatite and cellulose nanocrystals.

Based on the existing literature on bone cutting, silicone molds were used to fabricate rectangular block specimens with dimensions of 25 × 25 × 30 mm^3^ (Figure 1a). The specimens were prepared with and without nanocellulose (Figure 1b).

Standardized specimens, such as those used in tensile or compression tests, are not available for measuring the cutting force. Therefore, similar to previous bone-cutting tests [28], rectangular block specimens were used. The cutting tests were performed at a depth of 6 mm.

In the present study, the epoxy resin was reinforced with bioceramic HAP and CNCs to enhance its strength and stiffness, respectively [26]. Samples were prepared to compare differences in cutting forces resulting from these reinforcements. In addition to mechanical enhancements, potential applications of HAP and CNCs for bone regeneration were considered [29].

**Figure 2:**
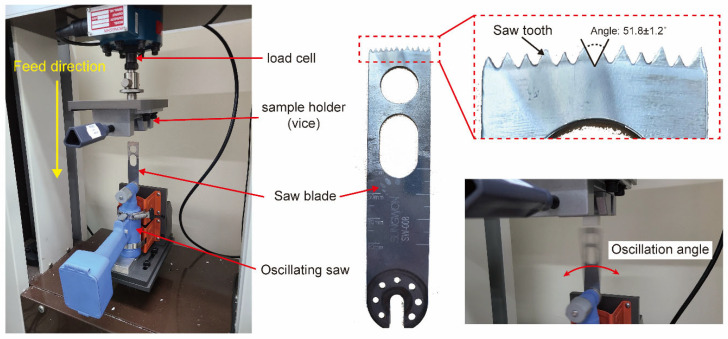
Setup for measuring the cutting force of composite materials with an oscillating saw.

The setup for measuring the sawing force consisted of a load cell and a sample holder positioned at the top, with an oscillating saw fixed at the bottom. The feed direction was configured such that the sample holder moved downward during the experiment. The saw tooth was triangular, with an angle of 51.8 ± 1.2°. During the operation, the saw blade moved within the range of the oscillation angle (Figure 2).

**Figure 3:**
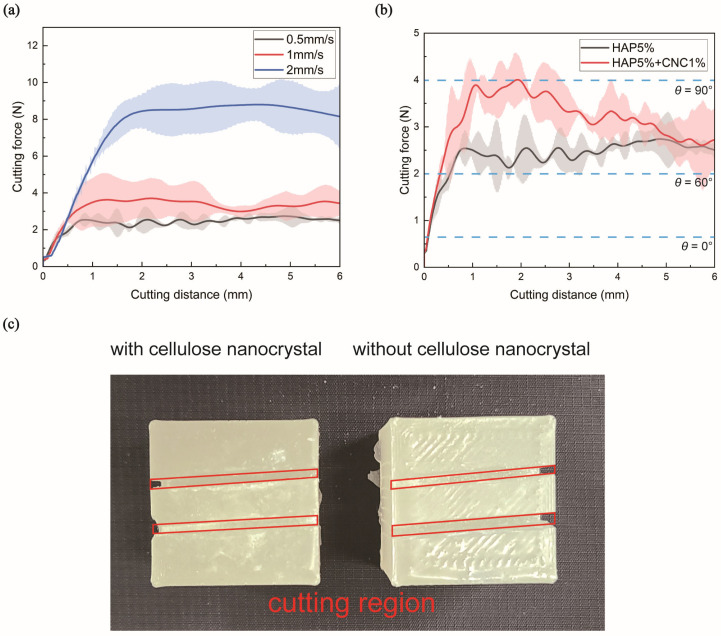
Measurement of the cutting force using an oscillating saw. (**a**) Cutting force results for the 5% hydroxyapatite (HAP) composite material based on the feed rate, including the average and standard deviation. (**b**) Comparison of cutting force results for the 5% HAP composite material and 5% HAP + 1% cellulose nanocrystal composite material based on the 0.5 mm/s feed rate, including the average cutting force and its standard deviation. (**c**) Appearance of the composite material after completion of cutting.

For the composite material containing only HAP, the cutting forces at the initial cutting distance of 2 mm were 2.344 ± 0.4507 N at 0.5 mm/s, 3.6513 ± 1.0321 N at 1 mm/s, and 8.4301 ± 0.8674 N at 2 mm/s (Figure 3a). In contrast, the composite material containing both HAP and CNCs exhibited cutting forces of 4.005 ± 0.5469 N at 0.5 mm/s (Figure 3b). For HAP, cutting forces increased with speed. For samples with CNC addition, a slightly higher cutting force was observed at the same cutting speed. This suggests that the initial cutting force for the composite material with CNCs was higher. Furthermore, the cutting speed used in this study matches that of previous research on actual animal bone cutting, demonstrating similar cutting force behavior [28,30]. For this reason, the addition of CNCs makes the composite material more brittle and prone to fragmentation, resulting in increased production of bone chip-like fragments and surface irregularities during cutting. This behavior aligns with the fracture toughness and SEM surface analysis presented later in this study. Figure 3c shows an image of the composite material after cutting, where the cut surface appears smooth, which is similar to real bone.

The results of the cutting-force measurement using an oscillating saw showed that the vertical cutting force was significantly lower, as stated in previous studies. Additionally, as the feed rate increased, the cutting force also increased, which is consistent with the behavior observed when cutting real bone [28].

The inclusion of CNCs led to a decrease in the cutting force as the feed rate increased and the saw cut deeper into the material. This is because uniformly distributed CNCs aid in cutting by promoting the propagation of microcracks, thereby continuously reducing the required force [31]. This phenomenon enables surgeons to practice more precisely on materials that mimic the varying thickness of real bone, thereby enhancing surgical training accuracy [32].

The cutting force was also found to align with the angle of the osteon in real knee bones perpendicular to the saw blade [33]. When the osteon is oriented at a 90° angle to the blade, the vertical cutting force at a cutting speed of 0.5 mm/s is approximately 4 N, which is similar to the force observed when CNCs are added to the composite material (Figure 3b). At a 0° angle to the blade, the vertical cutting force is approximately 0.5 N, and, at both 60° and 120° angles, the force is approximately 2 N [30].

In previous research, the stiffness of the epoxy was approximately 2 GPa, and, for HAP 5 wt%, it was approximately 2.6 GPa. However, when CNCs were added, the stiffness increased to approximately 4 GPa, approaching the reported elastic modulus of proximal femur bone, which is approximately 6–7.5 GPa [26,34]. This enhancement in stiffness is consistent with prior findings that adding CNCs increases stiffness while decreasing toughness in tensile tests, a trend also observed in the mechanical properties reflected due to differences in cutting force. Therefore, higher cutting forces are generated owing to increased stiffness and brittleness, causing greater plastic deformation during cutting. In summary, a mixture of 5% HAP and 1% CNCs in the composite material allows for conditions that closely mimic various bone-cutting scenarios [30,35].

**Figure 4:**
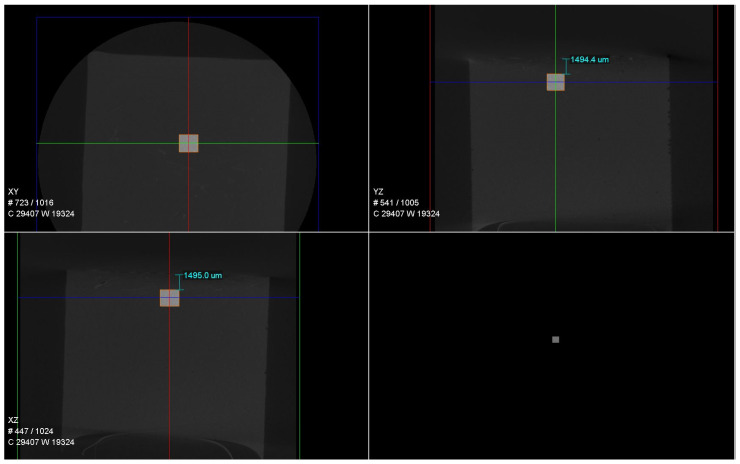
Results from XRM measurements. The brightly marked area indicates the high-resolution measurement region.

**Figure 5:**
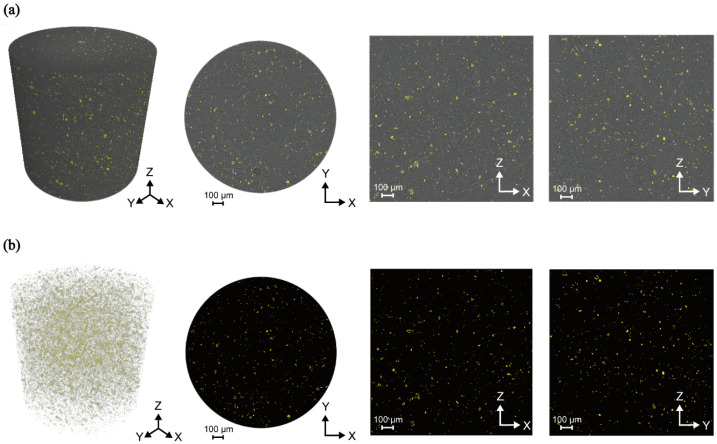
High-resolution results from XRM measurements. (**a**) A three-dimensional (3D) image illustrating the dispersion of nanocellulose within the epoxy matrix, along with views from various 3D planes. (**b**) An image depicting only the nanocellulose after the removal of the epoxy matrix.

Figure 4 shows the measurement locations where high-resolution measurements were taken in the cuboid specimen. The XRM results are shown in three dimensions, with positions indicated on the XY, YZ, and XZ planes. The bright gray areas indicate the measurement positions, as shown in Figure 5.

Recent studies on cortical bone thickness have found that the thickness of the proximal part near the cutting area during knee replacement surgery is 1.28 ± 0.4 mm for women and 1.74 ± 0.3 mm for men [36]. Therefore, the range for measuring CNCs with high resolution in the composite block was selected as 1.5–2 mm (Figure 4). Additionally, the 1.5–2 mm region shows the most significant differences in the cutting force (Figure 3b).

Using XRM, the cuboid specimen was positioned through preliminary measurements. High-resolution X-ray measurements were conducted using a cylindrical volume with a diameter of 1.5 mm and a height of 1.5 mm. The analyzed images confirmed the surface area distribution as 0.95 mm^2^ for 1 wt% of CNCs within the composite material across each plane, revealing a highly uniform dispersion.

The volume fraction of the CNCs was found to be 1.19%, which is consistent with the 1% weight percentage synthesized in the sample. Thus, it was confirmed that the composite material had a uniform distribution and the correct proportion of CNCs.

Additionally, during the synthesis, the distribution of CNCs was analyzed by calculating the area of CNCs at different depths. The numerical area values confirmed that the CNCs were uniformly distributed throughout the composite material. Therefore, the consistent distribution observed in the 3D X-ray images was corroborated by the numerical data, validating that the CNCs were evenly dispersed at various depths within the composite material.

**Figure 6:**
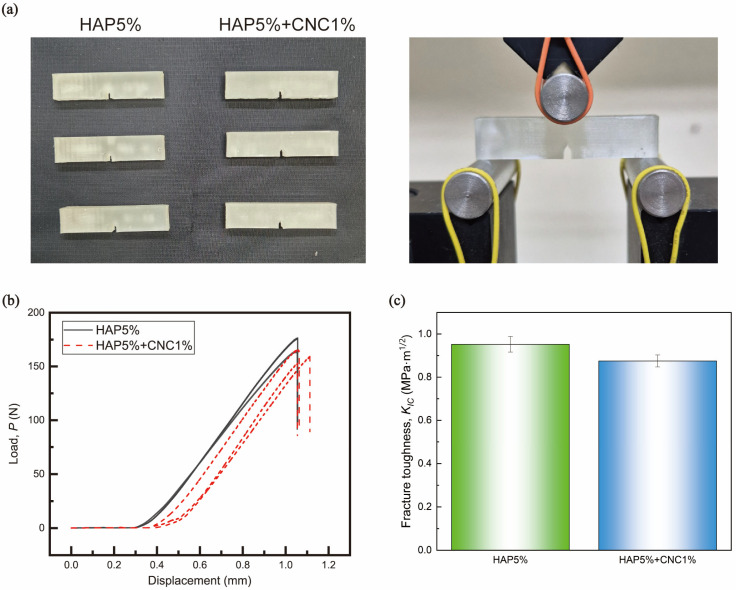
The three-point bending tests of the single-edge-notch bending specimens of HAP and HAP + cellulose nanocrystals (CNC) (**a**) and fracture toughness experimental setup. (**b**) Load-displacement curve. (**c**) Fracture toughness.

Fracture toughness was measured using the three-point bending method (Figure 6a). Load-displacement curves for both composite materials were measured using three-point bending on SENB samples. Both materials exhibited brittle characteristics, with the samples fracturing as crack propagation occurred. Fracture toughness was calculated using the following equation [37]:

(1) $$K_{IC}=\frac{P_{I}}{B\sqrt{W}}f\left(x\right)$$

(2) $$f\left(x\right)=6x^{1/2}\;\frac{\left[1.99-x\left(1-x\right)\left(2.15-3.93x+2.7x^{2}\right)\right]}{\left(1+2x\right){\left(1-x\right)}^{3/2}}$$

The results showed that, unlike differences in toughness reported in previous studies, there is no significant difference in fracture toughness between the materials. However, adding CNCs leads to a decrease in fracture toughness due to the material’s more brittle nature (Figure 6b,c) [26]. This difference is attributed to the characteristics of the oscillating saw, which causes irregular small cracks, resulting in greater cutting force in the vertical direction as the vibrating saw blade impacts the material [38]. Additionally, the reduced fracture toughness leads to easier fragmentation of the material, creating greater resistance and cutting force between the oscillating saw and the composite, similar to the generation of bone chips [30].

**Figure 7:**
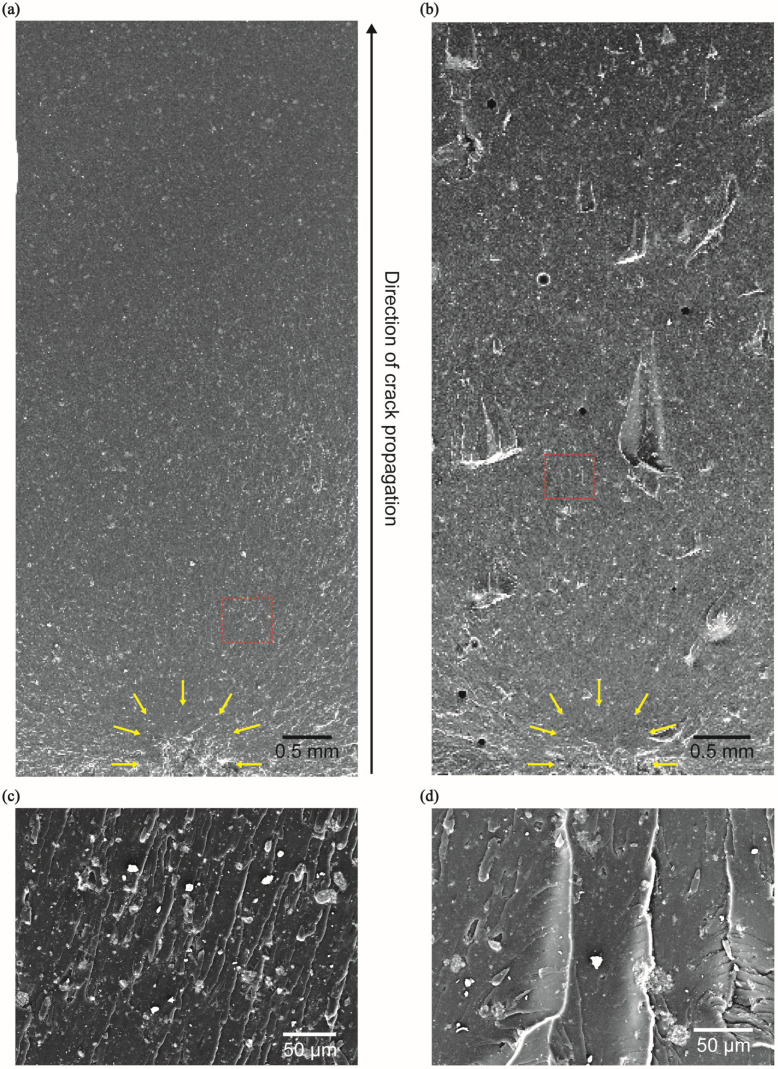
Scanning electron microscopy images of the fracture surface highlighting representative critical flaws, marked by yellow arrows. (**a**) HAP5%, (**b**) HAP5% + CNC1%. Magnified image (highlighted by the red square) of (**c**) HAP5%, (**d**) HAP5% + CNC1%.

Scanning electron microscopy analysis revealed that the surface of the HAP sample is smooth, with a clean fracture path following crack propagation (Figure 7a,c). However, in the HAP + CNC composite, irregular fracture surfaces and numerous fragmented particles can be observed (Figure 7b,d). These findings reflect the material characteristics identified earlier, wherein the oscillating saw generated high cutting force and the composite exhibited reduced fracture toughness. This behavior closely resembles the uneven surface observed in human bone fractures due to crack propagation [39].

**Figure 8:**
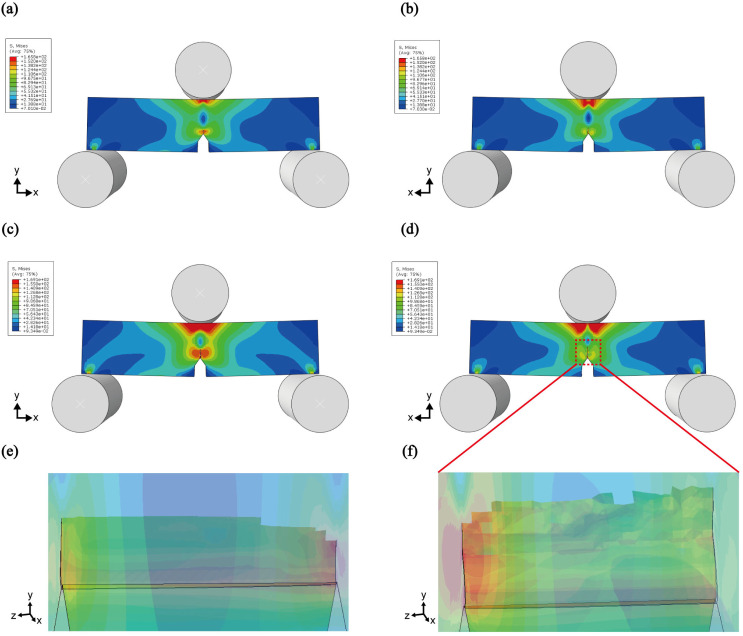
Finite element analysis of crack growth, (**a**,**b**) HAP5%, (**c**,**d**) HAP5% + CNC1%. Magnified image of crack propagation of (**e**) HAP5% and (**f**) HAP5% + CNC1%.

These characteristics can also be observed in the finite element analysis. When simulating the SENB specimen using the three-point bending method, crack propagation in the 5% HAP sample was found to be shorter due to its higher fracture toughness (Figure 8a,b). In contrast, the crack rapidly propagates in materials with lower fracture toughness and more brittle characteristics (Figure 8c,d). Additionally, as seen in the SEM images (Figure 7), the surface of the crack becomes more irregular and rougher when CNC is added, compared with when it is not (Figure 8e,f).

**Figure 9:**
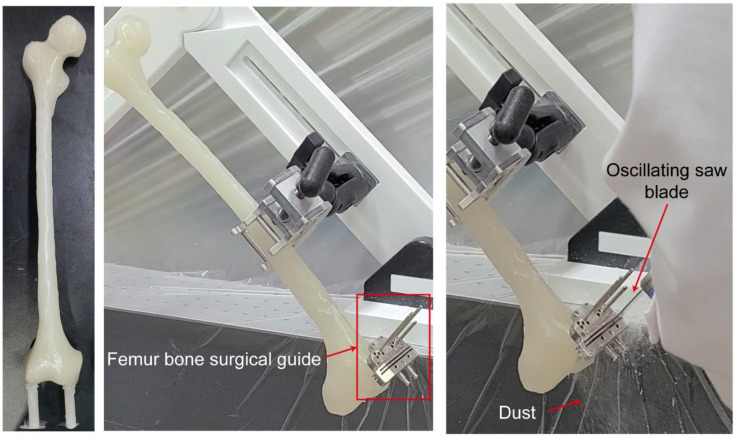
Application: fabrication and cutting test of the femur bone model.

As a result, femur bone-shaped specimens were created, and surgical guides were fixed. Cutting tests were performed using an oscillating saw, which confirmed that the composite material could be used for surgical training (Figure 9). This composite material, utilizing bioceramics and natural fibers, reduces the risks associated with debris during cadaver-based training while also providing bone-like mechanical properties [14]. It enhances the realism of surgical training by simulating the power and conditions of cutting real bones in a safe environment. This research aimed to provide a material that allows surgeons to safely and repeatedly practice bone cutting under realistic forces with consistent strength and tactile feedback, ultimately enhancing skill development.

Additionally, complex shapes can be fabricated in desired forms, allowing surgeons to practice using actual patient computed tomography data.

## 4. Conclusions

This study aimed to overcome the limitations of current training composites made from real bone and synthetic fibers by measuring the cutting force using an oscillating saw on a composite material. An experimental environment was created that was similar to that of bone, and it was confirmed that the addition of CNCs resulted in an increase in the initial cutting force, which was greater than that of the composite material with HAP. Additionally, the cutting force with nanocellulose closely matches the 4 N cutting force observed in real bone, where the osteon orientation was perpendicular to the cutting direction. This is because the uniformly distributed CNCs facilitated the cutting by influencing both fracture toughness and surface roughness. This study provides foundational data for measuring the cutting force on composite materials using an oscillating saw. Future research will focus on double casting with trabecular bone material to study changes in the cutting force with depth, with the aim of synthesizing materials that more closely mimic the cutting force of real bone. In addition, future research efforts will be directed to studying the impact of variations in saw blade size and shape on the cutting force.

## Figures and Tables

**Table 1:** Composite materials weight ratio.

Composite Materials	Resin	Hardener	HAP	CNC
HAP 5 wt%	71.25	23.75	5	-
HAP 5 wt% with CNC 1 wt%	70.5	23.5	5	1

HAP: hydroxyapatite; CNC: cellulose nanocrystals.

## Data Availability

The data presented in this study are available in the insert article.
